# Editorial: Filial piety as a universal construct: From cultural norms to psychological motivations

**DOI:** 10.3389/fpsyg.2022.980060

**Published:** 2022-08-16

**Authors:** Olwen Bedford, Kuang-Hui Yeh, Chee-Seng Tan

**Affiliations:** ^1^Department of Psychology, National Taiwan University, Taipei, Taiwan; ^2^Institute of Ethnology, Academia Sinica, Taipei, Taiwan; ^3^Department of Psychology and Counseling, Tunku Abdul Rahman University, Kampar, Malaysia

**Keywords:** filial piety, adolescent wellbeing, intergenerational relations, authoritarian parenting, self-esteem, adolescent development, parent-child relations, Chinese culture

Social science researchers often define *filial piety* as a set of norms, values, and practices regarding how children should behave toward their parents. In contrast, this Research Topic features a contextualized personality approach to the study of filial piety, the **Dual Filial Piety Model** (DFPM; Yeh and Bedford, 2003, 2004), which focuses on two universal human motivations in the context of the parent-child relationship: **reciprocal** filial piety (RFP: reflecting the need for interpersonal relatedness in terms of emotional safety and affective bonding with parents through expressions of love and affection) and **authoritarian** filial piety (AFP: reflecting the need for social belonging and collective identity by avoiding punishment and gaining social rewards through learning to obey parental demands and social norms). The focus on universal motivations extends the scope of filial piety research from Confucian societies to cross-cultural investigations and encompasses domains from adolescent development and psychosocial adjustment to intergenerational relations, population aging, and eldercare.

The 10 articles in this Research Topic demonstrate this scope by investigating four interrelated themes (1) mechanisms of parent-child interaction, (2) individual socio-emotional development and adaptation, (3) the link between individual differences in filial piety and the wider socio-demographic context, and (4) cross-cultural/national comparisons. Five articles investigated Chinese societies: Wu and Yeh examined the strategies Taiwanese adolescents use to practice filial piety when in conflict with their parents. Their findings broaden the understanding of filial piety in modern Chinese societies and have implications for adolescents' wellbeing and family life. Lin and Wang explored the roles of parenting style and social ecology in the development of filial piety and examined all possible bidirectional paths with longitudinal data from China. Their work bridged filial piety and mainstream parenting theory and provided a comprehensive picture of parent-child interaction within the DFPM. Guo M. et al. demonstrated the differential role of the two filial motivations in mediating the relationships of parental autonomy support and parental control to academic autonomous motivation for adolescents in China. Guo X. et al. investigated the mechanism linking filial piety motivation and early adolescent psychosocial development by focusing on the unique effects of each aspect of filial piety on academic achievement and subjective wellbeing for adolescents in China. Pan and Tang found that for adolescents in China, RFP alleviated depressive symptoms by enhancing cognitive autonomy and reducing academic pressure, whereas AFP exacerbated depressive symptoms by hampering cognitive autonomy and increasing academic pressure.

Five articles extended the DFPM to non-Confucian societies: Zheng et al. applied the DFPM to moral psychology and culture (China and Indonesia). They examined four psychological mediators in the link between dual filial piety and altruistic behavior, as well as the moderating effects of nation on each mediating path (an alternative method of comparing cultures). Both types of filial piety facilitated development of prosocial behavior through different mediating paths conditioned by culture. Qiao et al. found that filial piety has an indirect relationship with moral disengagement through the dark triad personality among university students from Chinese and Islamic societies. Machiavellianism mediated the negative relationship between RFP and moral disengagement, and narcissism mediated the positive relationship between AFP and disengagement. Nainee et al. applied the Malaysian version of the Dual Filial Piety Scale (DFPS) to elucidate the relationships among parenting style, filial piety, and life satisfaction for adolescents in Malaysia. The results highlighted the benefits of culture-infused parenting. Tran et al. translated the DFPS into Polish and tested its psychometric properties. They demonstrated the factor structure invariance of the scale across gender and student-employee groups and the anticipated gender difference in attitudes. This work extends the cross-cultural validity of the DFPM to Eastern Europe, and provides a counterpoint the standard paradigm of East-West differences. Lim et al. applied an English version of the DFPS to Asian and Caucasian Americans. Their work demonstrated the cross-cultural applicability of the model and the potential to reflect significant filial differences within an individualist society.

In sum, these 10 studies covered diverse samples from different cultures with four different language versions of the DFPS (Chinese, English, Polish, and Malay) to demonstrate theoretically consistent results that broaden the understanding of filial piety in modern societies. These studies provide evidence to support the rationale for redefining filial piety as a universal psychological construct, and also overturn some cultural stigmas associated with the traditional conceptualization of Chinese filial piety by demonstrating positive implications of filial piety through the DFPM perspective.

## Author contributions

OB, K-HY, and C-ST contributed to summarizing the articles in the topic and writing the editorial. All authors contributed to the article and approved the submitted version.

## Conflict of interest

The authors declare that the research was conducted in the absence of any commercial or financial relationships that could be construed as a potential conflict of interest.

## Publisher's note

All claims expressed in this article are solely those of the authors and do not necessarily represent those of their affiliated organizations, or those of the publisher, the editors and the reviewers. Any product that may be evaluated in this article, or claim that may be made by its manufacturer, is not guaranteed or endorsed by the publisher.
